# Discovery and Validation of Nitroxoline as a Novel STAT3 Inhibitor in Drug-resistant Urothelial Bladder Cancer

**DOI:** 10.7150/ijbs.63125

**Published:** 2021-07-25

**Authors:** Wenfeng Lin, Jingkai Sun, Takuya Sadahira, Naijin Xu, Koichiro Wada, Chunxiao Liu, Motoo Araki, Abai Xu, Masami Watanabe, Yasutomo Nasu, Peng Huang

**Affiliations:** 1 Department of Urology, Okayama University Graduate School of Medicine, Dentistry and Pharmaceutical Sciences, Okayama, Japan; 2 Department of Urology, Zhujiang Hospital, Southern Medical University, Guangzhou, China; 3 Department of Urology, Sir Run Run Shaw Hospital, Zhejiang University School of Medicine, Hangzhou, China; 4 Center for Innovative Clinical Medicine, Okayama University Hospital, Okayama, Japan; 5 Okayama Medical Innovation Center, Okayama University, Okayama, Japan

**Keywords:** Urothelial bladder cancer, doxorubicin, cisplatin, chemoresistance, nitroxoline, STAT3.

## Abstract

Repeated cycles of first-line chemotherapy drugs such as doxorubicin (DOX) and cisplatin (CIS) trigger frequent chemoresistance in recurrent urothelial bladder cancer (UBC). Nitroxoline (NTX), an antibiotic to treat urinary tract infections, has been recently repurposed for cancer treatment. Here we aimed to investigate whether NTX suppresses drug-resistant UBC and its molecular mechanism. The drug-resistant cell lines T24/DOX and T24/CIS were established by continual exposure of parental cell line T24 to DOX and CIS, respectively. T24/DOX and T24/CIS cells were resistant to DOX and CIS, respectively, but they were sensitive to NTX time- and dose-dependently. Overexpressions of STAT3 and P-glycoprotein (P-gp) were identified in T24/DOX and T24/CIS, which could be reversed by NTX. Western blot revealed that NTX downregulated p-STAT3, c-Myc, Cyclin D1, CDK4, CDK6, Bcl-xL, Mcl-1, and Survivin, which were further confirmed by Stattic, a selective STAT3 inhibitor. In vivo, NTX exhibited the significant anti-tumor effect in T24/DOX and T24/CIS tumor-bearing mice. These results suggested that NTX-induced P-gp reversal, G0/G1 arrest, and apoptosis in drug-resistant UBC were mediated by inhibition of STAT3 signaling. Our findings repurpose NTX as a novel STAT3 inhibitor to induce P-gp reversal, G0/G1 arrest, and apoptosis in drug-resistant UBC.

## Introduction

Urothelial bladder cancer (UBC) is a malignant tumor originating primarily from the transitional cells of bladder urothelium 1. The 2021 Cancer Statistics 2 reported an estimation of 83,730 new UBC cases and 17,200 UBC-specific deaths in America. Moreover, 50%-70% patients with non-muscle-invasive bladder cancer (NMIBC) will suffer a relapse following transurethral resection, while approximately 30% will progress into muscle-invasive bladder cancer (MIBC), mainly causing UBC-specific deaths 3. Both doxorubicin (DOX/ADM/ADR) and cisplatin (CIS/DDP/CDDP) have been the first-line chemotherapy drugs effectively against UBC, but the high rates of DOX- and CIS- chemoresistance in recurrent UBC remain a major barrier to improve the prognosis of patients 4, 5. It still maintains a challenge to explore novel effective chemotherapeutic agents for the treatment of drug-resistant UBC.

Multidrug resistance (MDR), a clinical obstacle to the long-term success in chemotherapy, represents the cross-resistance of tumor cells to various chemotherapeutic agents with different structures and functions 6. One of the main reasons for MDR phenotype is the overexpressed 170-kDa transmembrane glycoprotein, P-glycoprotein (P-gp). It is encoded by the ATP-binding cassette B1 (ABCB1) gene, with an alternative name as multidrug resistance 1 (MDR1) 7. As a drug efflux pump, P-gp extrudes its substrates such as DOX out of tumor cells, thus resulting in a reduction of intracellular drug concentration 8. P-gp has also been reported to reduce the tumor sensitivity to the non-P-gp substrates such as CIS, while inhibition of P-gp has the potential to increase CIS sensitivity 9, 10. Signal transducer and activators of transcription 3 (STAT3) acts as a crucial transcription factor to regulate its downstream target genes, which subsequently involves in diverse biological processes such as cell proliferation, survival, and chemoresistance 11. Overexpression and activation of STAT3 correlate with the poor prognosis of UBC 12, 13, and STAT3 requires the phosphorylation at the residues of Tyr705 (p-STAT3 (Y705)) and Ser727 (p-STAT3 (S727)) for its maximal activation 14. The chromatin immunoprecipitation (ChIP) assay revealed the potential binding of STAT3 to MDR1 gene promoter region, and increasing evidence suggests that inhibiting STAT3 activation effectively downregulates the expression of P-gp 15, 16. Based on the above research evidence, the development of potent inhibitors targeting STAT3 signaling may be a promising therapeutic strategy against drug-resistant UBC. Although multiple inhibitors targeting STAT3 in cancer have been identified in preclinical and early phase clinical trials, the potential low efficacy or adverse effects may limit their clinical transformation 17.

Drug repurposing refers to discovering novel indications of the currently marketed drugs which have been applied to treat other diseases 18. Nitroxoline (NTX), an antibiotic used clinically to treat urinary tract infections, has been repurposed for the treatment of multiple tumors such as UBC 19-21, pancreatic cancer 22, and glioma 23. Several studies have reported its anti-tumor activity against UBC since Shim and coworkers firstly identified NTX as an antiangiogenic agent in UBC through inhibition of methionine aminopeptidase-2 (MetAP2) 19-21. In an orthotopic mouse model, NTX was demonstrated to exhibit anti-UBC effect with favorable safety profile and pharmacokinetic properties 21. Our previous study has also revealed that NTX suppresses the progression of UBC by reversing epithelial-mesenchymal transition (EMT) and enhancing anti-tumor immunity 20. Prior studies have noted that P-gp expression is identified in pre-chemotherapy UBC tissue samples, which increases with MDR after chemotherapy 24, 25.

However, it has not been reported whether NTX and its analogues suppress the growth of drug-resistant UBC. And the mechanism whether NTX inhibits STAT3 signaling and reverses P-gp overexpression, remains to be explored at the molecular level. Drug repurposing of NTX in drug-resistant UBC may contribute to the following advantages including its well-known drug properties (pharmacokinetics, efficacy, toxicity and drug interactions), developing a putative agent with fewer funds and shorter period 21. Both in vitro and in vivo experiments were performed to investigate whether NTX could reverse P-gp overexpression and effectively inhibit the growth of drug-resistant UBC cell lines through STAT3 signaling regulation.

## Material and Methods

### Chemicals and antibodies

The chemicals including DOX and CIS were purchased from MedChemExpress (MCE, New Jersey, USA). Both DOX and CIS were dissolved in phosphate buffered saline (PBS), followed by ultrasonic bath and 0.22-µm filtration. Nitroxoline (NTX) was provided by Jiangsu Asieris Pharmaceuticals in China. NTX was treated as PBS dissolution with 0.22-µm filtration. Stattic, a selective inhibitor of STAT3 activation and dimerization, was obtained from Tocris Bioscience (#2978, Bristol, UK), which was treated as dimethyl sulfoxide (DMSO) dissolution with a 20-mM stock solution. Primary antibodies against β-Actin (#4970), STAT3 (#12640), p-STAT3 (Y705) (#9145), p-STAT3 (S727) (#9134), MDR1/P-gp (#13342), c-Myc (#5605), Cyclin D1 (#2978), CDK4 (#2906), CDK6 (#3136), Bcl-xL (#2764), Mcl-1 (#4572), and Survivin (#2808) were provided by Cell Signaling Technology (CST, USA). Anti-rabbit IgG (#7074) and anti-mouse IgG (#7076) were the corresponding secondary antibodies.

### Cell culture of drug-resistant UBC cell lines

Human UBC cell line T24 was purchased from the American Type Culture Collection (ATCC, USA). DOX-resistant bladder cancer cell line, T24/DOX, and CIS-resistant bladder cancer cell line, T24/CIS, were established from our laboratory based on continual exposure of parental cell line T24 to the culture media with DOX and CIS, respectively 26-28. T24, T24/DOX, and T24/CIS cells were incubated in DMEM medium containing 10% FBS, 1% penicillin-streptomycin (Invitrogen, USA). To maintain the drug-resistant characteristics, T24/DOX cells were cultured with 1 μM DOX while T24/CIS cells cultured with 10 μM CIS prior to experiment.

### Cell viability assay

T24, T24/DOX, and T24/CIS (2 × 10^3^ /well) in 96-well plates were allowed to attach overnight. For drug sensitivity analysis, T24 and T24/DOX cells were treated with DOX (0, 0.1, 1, 10, 20, and 100 μM) for 24 h, while T24 and T24/CIS cells underwent 24 h treatment with CIS (0, 10, 20, 40, and 80 μM). To determine the effect of NTX on cell proliferation, T24, T24/DOX, and T24/CIS cells were exposed to NTX (0, 2.5, 5, 10, 20, and 40 μM) for 24, 48, and 72 h. At the indicated time points, culture media were removed and the cells were washed gently with PBS. The prepared XTT working solution (Cell Proliferation Kit II, #11465015001, Sigma-Aldrich) was added to the cells, which were subsequently incubated for 4-24 h at 37 ℃. Finally, the optical density (OD) levels were determined by the Model 680 Microplate Reader (Bio-Rad, USA).

### Hoechst 33342 staining

T24, T24/DOX, and T24/CIS (2 × 10^5^ /well) in 6-well plates firstly underwent 24 h culture. Next, after 48 h treatment with 0 and 40 μM NTX, cells were stained at room temperature with 5 µg/ml Hoechst 33342 (#H3570, ThermoFisher Scientific, USA) for 20 min. The occurrence of apoptotic cells was observed under fluorescence microscope.

### Cell cycle distribution analysis

T24, T24/DOX, and T24/CIS (2 × 10^5^ /well) were seeded in 6-well plates. After 24 h incubation with NTX (0, 10, 20, or 40 µM), cells were rinsed twice with PBS and fixed in precooling 70% ethanol for over 18 h at 4℃. Subsequently, they were rinsed twice with PBS and stain buffer (BD Biosciences, #554656). Following by 15 min staining in PI/RNase buffer (BD Biosciences, #550825), the analysis was performed by MACSQuant Analyzer 10 and MACSQuantify^TM^ Software 2.6.

### Apoptosis examination

T24, T24/DOX, and T24/CIS (1.5 × 10^5^ /well) in 6-well plates underwent 48 h incubation of NTX. After twice rinsing, they underwent 1X Annexin V binding buffer resuspension to obtain a density of 1 × 10^6^ cells/ml. In the dark, cell suspension (100 μl) underwent 15 min treatment with FITC Annexin V (5 µl) and PI (5 µl) (BD Biosciences, #556547). Finally, apoptosis was examined by MACSQuant Analyzer 10 and analyzed by MACSQuantify^TM^ Software 2.6.

### Western blot analysis

Total protein extraction was conducted with M-PER^TM^ Mammalian Protein Extraction Reagent (#78501, ThermoFisher Scientific, USA) and then quantified by Bradford protein assay. The same amount of protein (10 μg/well) was separated by 10% or 12% Mini-PROTEAN Gels and then transferred to 0.2 µm PVDF Transfer Packs (#1704156, Bio-Rad, USA) on the Trans-Blot Turbo transfer system (#170-4155, Bio-Rad, USA). After 1.5 h bovine serum albumin or non-fat milk blocking, and 4°C overnight incubation with primary antibodies, membranes underwent TBST washing. At room temperature, the secondary antibody was diluted to treat membranes for 1.5 h. The ECL detection reagent (#RPN2232, GE Healthcare) and ChemiDoc Imaging System (Bio-Rad, USA) were used to visualize proteins.

### Subcutaneous xenograft models of T24/DOX and T24/CIS

Male BALB/c nude mice (five-week-old) kept in specific pathogen-free (SPF) conditions were from Japan SLC, Inc. (Shizuoka, Japan). The mice experimentation was conducted in compliance with the Animal Care and Use Committee, Okayama University.

T24/DOX cells (1×10^6^) or T24/CIS cells (1×10^6^) in 100 µl resuspension, mixing Matrigel (BD Biosciences, USA) and Hank's buffered saline solution (HBSS) at 1:1, were used for subcutaneous inoculation to mouse right flank. The mice bearing about 100 mm^3^ T24/DOX tumor received two-week oral administration of NTX (40 mg/kg) or PBS (n = 6 per group) once per day. It was the same with T24/CIS tumor-bearing mice. Both mouse body weight and tumor growth were measured twice a week. Surgical resection, weighing, and photography of T24/DOX and T24/CIS tumors were conducted at the indicated endpoint. The formula of volume = 0.52 × length × width^2^, was applied to calculate tumor growth. T-PER Tissue Protein Extraction Reagent (#78510, ThermoFisher Scientific, USA) was used for extracting tumor tissue protein, which was subsequently analyzed by western blot. For histological examination, T24/DOX and T24/CIS tumors underwent 10% formaldehyde fixation, paraffin imbedding, and 4 μm section cut. Hematoxylin and eosin (H&E) were applied to stain the sections, followed by observing NTX-induced morphological alterations.

### Statistical analysis

GraphPad Prism 8.3 was used to perform statistical analyses including half maximal inhibitory concentration (IC50) calculation and statistical charts creation. Between-group difference was assessed by Student's t-test or one-way analysis of variance (ANOVA), with data expressing as mean ± standard deviation (SD).

## Results

### Characterization of DOX-resistant and CIS-resistant bladder cancer cell lines

**Figure 1:**
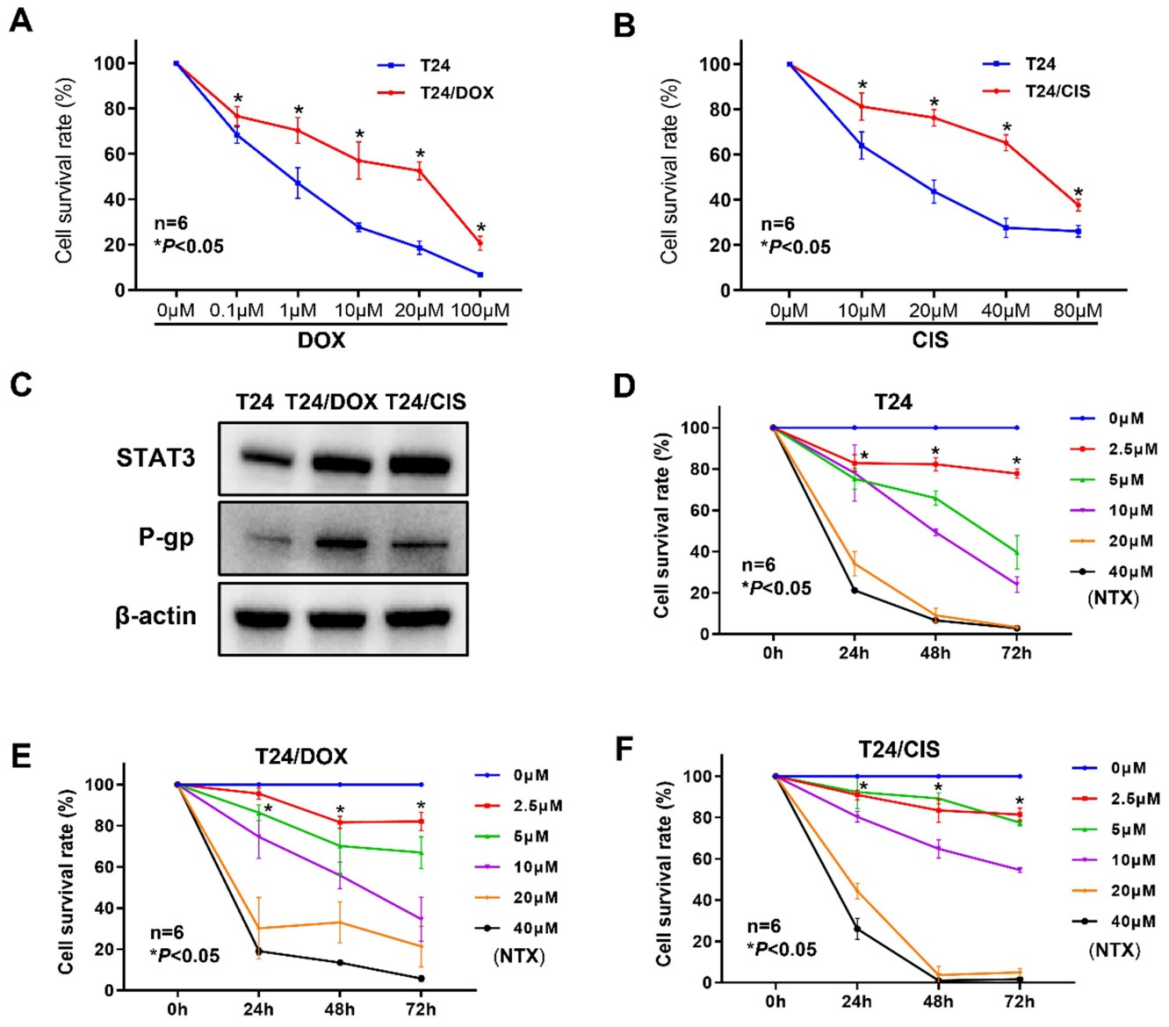
** T24/DOX and T24/CIS cells were resistant to DOX and CIS, respectively, but sensitive to NTX. (A)** T24 and T24/DOX cells were treated with different concentrations of DOX (0, 0.1, 1, 10, 20, or 100 μM) for 24 h, followed by the evaluation of cell viability using XTT assay. **(B)** T24 and T24/CIS cells were treated with different concentrations of CIS (0, 10, 20, 40, or 80 μM) for 24 h, followed by the evaluation of cell viability using XTT assay. Data are expressed as the mean ± SD (n=6). *P < 0.05 vs T24 parental cells. **(C)** The expression levels of STAT3 and P-gp in T24, T24/DOX, and T24/CIS cells were analyzed by western blot analysis. β-actin was used as a loading control. **(D)** T24, **(E)** T24/DOX, and **(F)** T24/CIS cells were exposed to different concentrations of NTX (0, 2.5, 5, 10, 20, and 40 μM) for 24, 48, and 72 h. The cell viability was determined by XTT assay. Data are shown as the mean ± SD (n=6). *P < 0.05 vs the control group.

To confirm whether bladder cancer cell lines acquired DOX or CIS resistance, the viability of T24/DOX or T24/CIS cells was determined by XTT assay following the indicated treatment for 24 h. Although DOX dose-dependently inhibited the proliferation of T24/DOX cells, the sensitivity to DOX was lower than that of parental T24 cells (**Figure 1A**). For DOX, the IC50 value in T24 cells was 0.71 μM [95% confidence interval (95%CI), 0.56-0.89], while that in T24/DOX cells was 10.93 μM (95%CI, 6.88-17.55), with a 15.4-fold change in IC50 (**Table 1**). Similarly, CIS dose-dependently inhibited the proliferation of T24/CIS cells, but the sensitivity to CIS was lower than that of parental T24 cells (**Figure 1B**). For CIS, the IC50 value in T24 cells was 16.27 μM (95%CI, 13.29-19.11), while that in T24/CIS cells was 58.04 μM (95%CI, 51.11-67.55), with a 3.6-fold change in IC50 (**Table 1**).

We next analyzed the expressions of STAT3 and P-gp by western blot. In **Figure 1C**, the levels of STAT3 and P-gp were upregulated in T24/DOX and T24/CIS cells compared with T24 parental cells.

### NTX inhibits the proliferation of DOX-resistant and CIS-resistant bladder cancer cells

The dose- and time-dependent viability inhibitions were observed in T24 parental cells (**Figure 1D**), and drug-resistant cell lines T24/DOX (**Figure 1E**), T24/CIS (**Figure 1F**). Moreover, for 48 h treatment of NTX, the IC50 values in T24/DOX (10.69 μM) and T24/CIS (11.20 μM) were slightly higher than that in T24 cells (7.85 μM), but IC50 values of NTX were not significantly different between T24 parental cells and drug-resistant cells (**Table 1**).

### NTX triggers G0/G1 phase arrest in DOX-resistant and CIS-resistant bladder cancer cells

**Figure 2:**
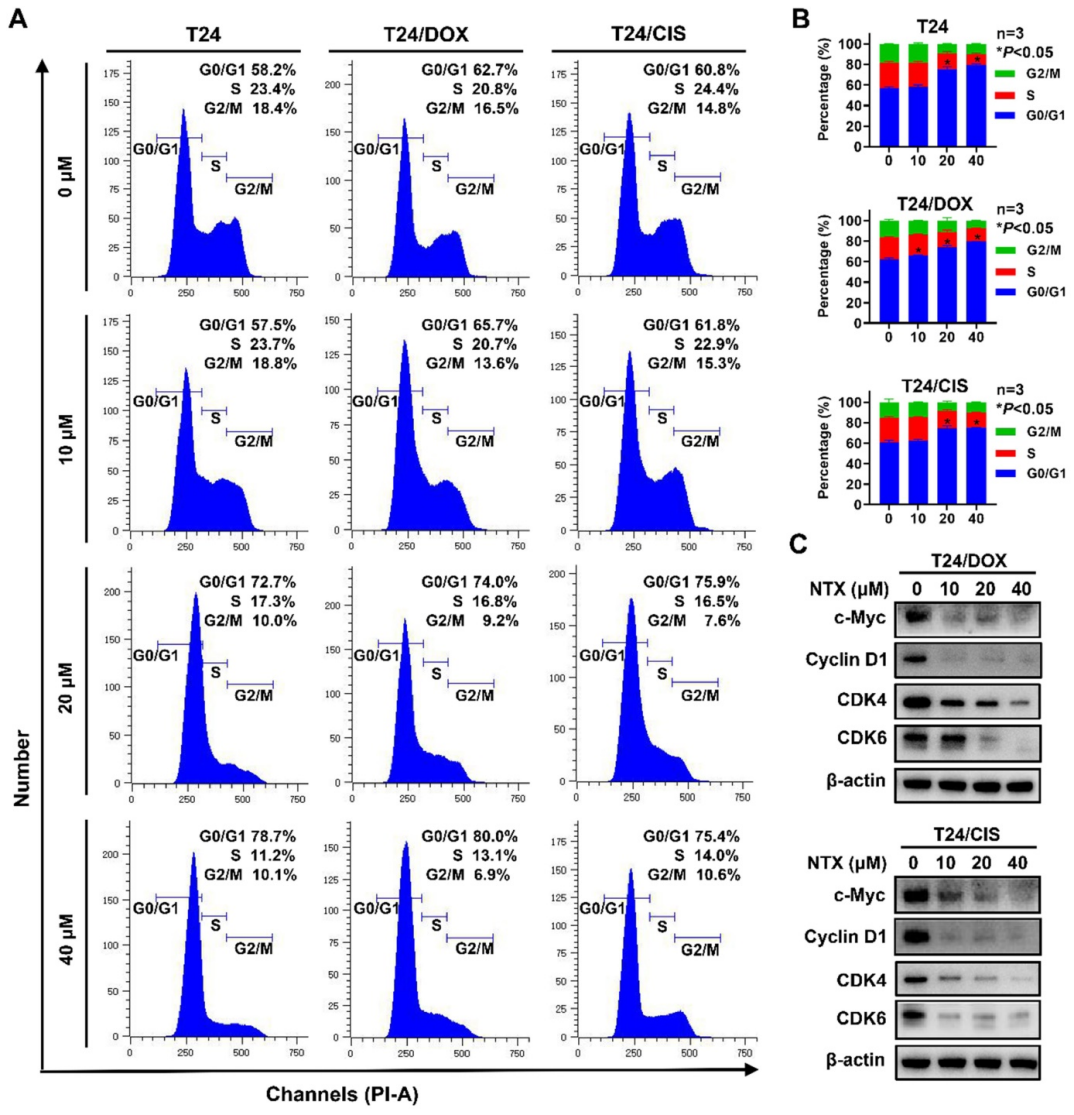
** NTX triggers G0/G1 phase cell cycle arrest in T24, T24/DOX, and T24/CIS cells. (A)** T24, T24/DOX, and T24/CIS cells were treated with the indicated concentrations of NTX (0, 10, 20, and 40 μM) for 24 h. The harvested cells were incubated with PI/RNase, followed by flow cytometric analysis of cell cycle distribution. **(B)** The proportions of cells in each phase (G0/G1, S, and G2/M) are presented in the histograms. Data are expressed as the mean ± SD from three independent experiments. *P < 0.05 vs the control group. **(C)** T24/DOX and T24/CIS cells were treated with NTX (0, 10, 20, and 40 μM) for 48 h. Proteins were extracted and western blot was used to analyze the expressions of c-Myc, Cyclin D1, CDK4, and CDK6. β-actin was used as a loading control.

To examine whether NTX inhibited the cell cycle progression, T24, T24/DOX, and T24/CIS cells were treated with different concentrations of NTX for 24 h, followed by flow cytometric analysis (**Figure 2A**). As drug concentration increased, a higher percentage of cells stayed at G0/G1 phase, while a lower percentage of cells stayed at the phases of S and G2/M in T24, T24/DOX, and T24/CIS cells (**Figure 2B**). NTX negatively regulated the G0/G1-related protein expressions (c-Myc, Cyclin D1, CDK4, and CDK6) in T24/DOX and T24/CIS cells (**Figure 2C**).

### NTX promotes apoptosis of DOX-resistant and CIS-resistant bladder cancer cells

**Figure 3:**
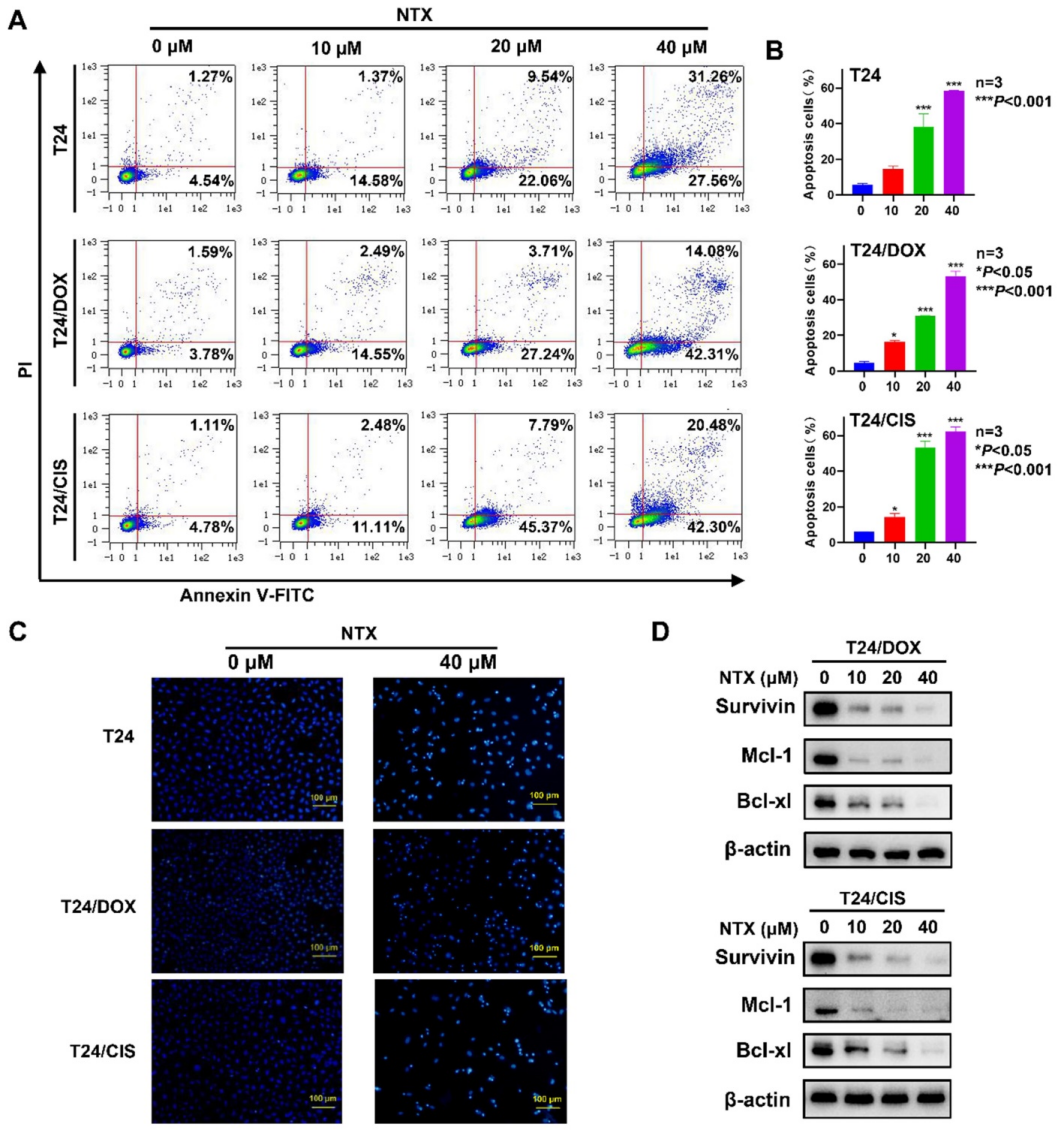
** NTX induces apoptosis of T24, T24/DOX, and T24/CIS cells. (A)** T24, T24/DOX, and T24/CIS cells were treated with the indicated concentrations of NTX (0, 10, 20, and 40 μM) for 48 h. The cells were stained with Annexin V-FITC/PI and flow cytometry was used to analyze the apoptotic rates.** (B)** The percentages of apoptotic cells are shown in the histograms. Data are expressed as the mean ± SD from three independent experiments. *P < 0.05, ***P < 0.001 vs the control group. **(C)** The indicated concentrations of NTX (0 and 40 μM) were used to treat T24, T24/DOX, and T24/CIS cells for 48 h. Hoechst 33342 was then used to stain the cell nuclei (blue), followed by fluorescence microscopy. Scale bar = 100 μm. **(D)** T24/DOX and T24/CIS cells were treated with NTX (0, 10, 20, and 40 μM) for 48 h, followed by western blot to assess the expressions of Bcl-xL, Mcl-1, and Survivin. β-actin was used as a loading control.

T24, T24/DOX, and T24/CIS cells were treated with NTX for 48 h, followed by flow cytometry (**Figure 3A**). The apoptotic rates of T24 cells in NTX groups were (14.65±1.47)%, (38.26±7.21)%, and (58.35±0.43)% of the total cells, compared with only (5.96±0.58)% in control group (**Figure 3B**). As NTX concentration increased, the apoptotic rates of T24/DOX cells were (4.42±0.92)%, (16.52±0.48)%, (30.84±0.17)%, and (53.37±2.67)%, while the rates of T24/CIS cells were (6.05±0.14)%, (14.49±1.89)%, (53.19±3.68)%, and (62.36±2.51)%, respectively (**Figure 3B**). Consistent with the apoptosis trend detected by flow cytometry, Hoechst staining results indicated a higher proportion of apoptotic T24, T24/DOX, and T24/CIS cells in NTX-treated groups, exhibiting such typical morphological changes as chromatin condensation and nuclear fragmentations (**Figure 3C**).

Western blot analysis revealed that the levels of Bcl-xL, Mcl-1, and Survivin decreased following NTX treatment (**Figure 3D**). Our results indicated that NTX promoted the apoptosis of both DOX-resistant and CIS-resistant T24 cells.

### NTX suppresses the STAT3 signaling and P-gp in T24/DOX and T24/CIS cells

**Figure 4:**
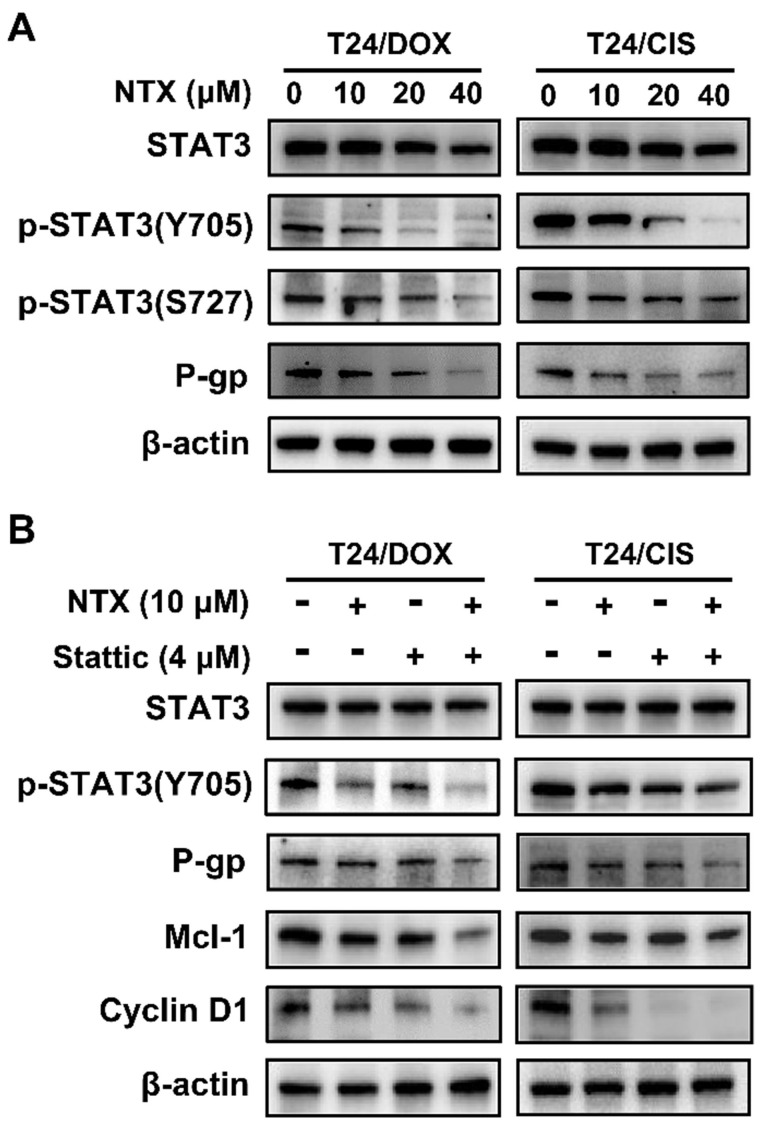
** NTX suppresses the STAT3 signaling pathway in T24/DOX and T24/CIS cells. (A)** Western blot was used to assess the expressions of STAT3, p-STAT3 (Y705), p-STAT3 (S727), and P-gp in T24, T24/DOX, and T24/CIS cells after they were treated with NTX (0, 10, 20, and 40 μM) for 48 h.** (B)** T24/DOX and T24/CIS cells were exposed to 10 μM NTX in the presence or absence of 4 μM Stattic for 24 h. Western blot was then performed to detect the expressions of STAT3, p-STAT3 (Y705), P-gp, Cyclin D1, and Mcl-1.

Western blot was applied to analyze whether NTX treatment regulated the expressions of proteins associated with STAT3 signaling and P-gp. **Figure 4A** illustrates that NTX significantly decreased the levels of STAT3, p-STAT3 (Y705), p-STAT3 (S727), and P-gp dose-dependently.

To further verify whether NTX induced apoptosis and G0/G1 arrest through the STAT3 signaling, T24/DOX and T24/CIS cells were incubated with NTX (10 μM) or Stattic (4 μM) for 24 h. Western blot analysis demonstrated that NTX (10 μM, 24h or 48 h) or Stattic (4 μM, 24h) alone did not significantly inhibit STAT3 expression, but higher concentrations of NTX (20 and 40 μM) decreased the levels of STAT3 (**Figure 4A, B**). The combination of NTX and Stattic synergistically downregulated the expressions of p-STAT3 (Y705), P-gp, Cyclin D1, and Mcl-1 (**Figure 4B**). These findings suggested that NTX could inhibit P-gp, promote apoptosis and G0/G1 arrest in DOX-resistant and CIS-resistant T24 cell lines via the STAT3 pathway.

### NTX exhibits the anti-tumor effect in T24/DOX and T24/CIS tumor-bearing mice

**Figure 5:**
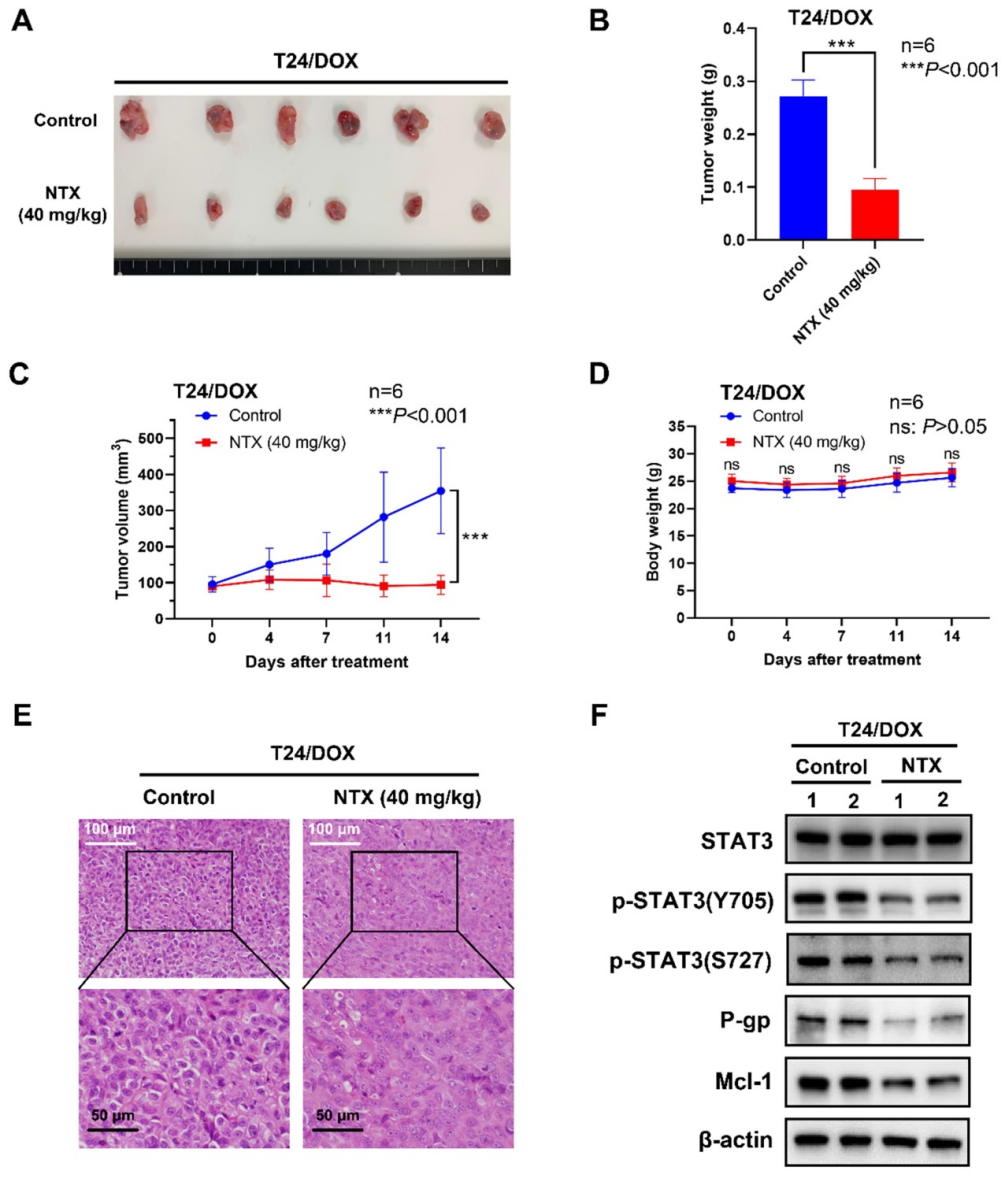
** NTX exhibits the anti-tumor effect on T24/DOX in vivo. (A)** T24/DOX tumors were excised from mice and photographed after two-week treatment.** (B)** The average tumor weight of NTX treatment group significantly decreased compared with that of vehicle group.** (C)** The growth curves of T24/DOX tumors at the indicated days following NTX treatment. **(D)** No significant difference was found in the body weight of T24/DOX tumor-bearing mice between treatment group and vehicle group. Data are expressed as the mean ± SD. ***P < 0.001, ns: *P* > 0.05, vs the vehicle group. **(E)** H&E staining was performed to observe the morphological alterations in T24/DOX tumors after NTX treatment. White scale bar = 100 μm; Black scale bar = 50 μm.** (F)** Proteins were extracted from T24/DOX tumors and western blot was used to detect the expressions of STAT3, p-STAT3 (Y705), p-STAT3 (S727), P-gp, and Mcl-1.

**Figure 6:**
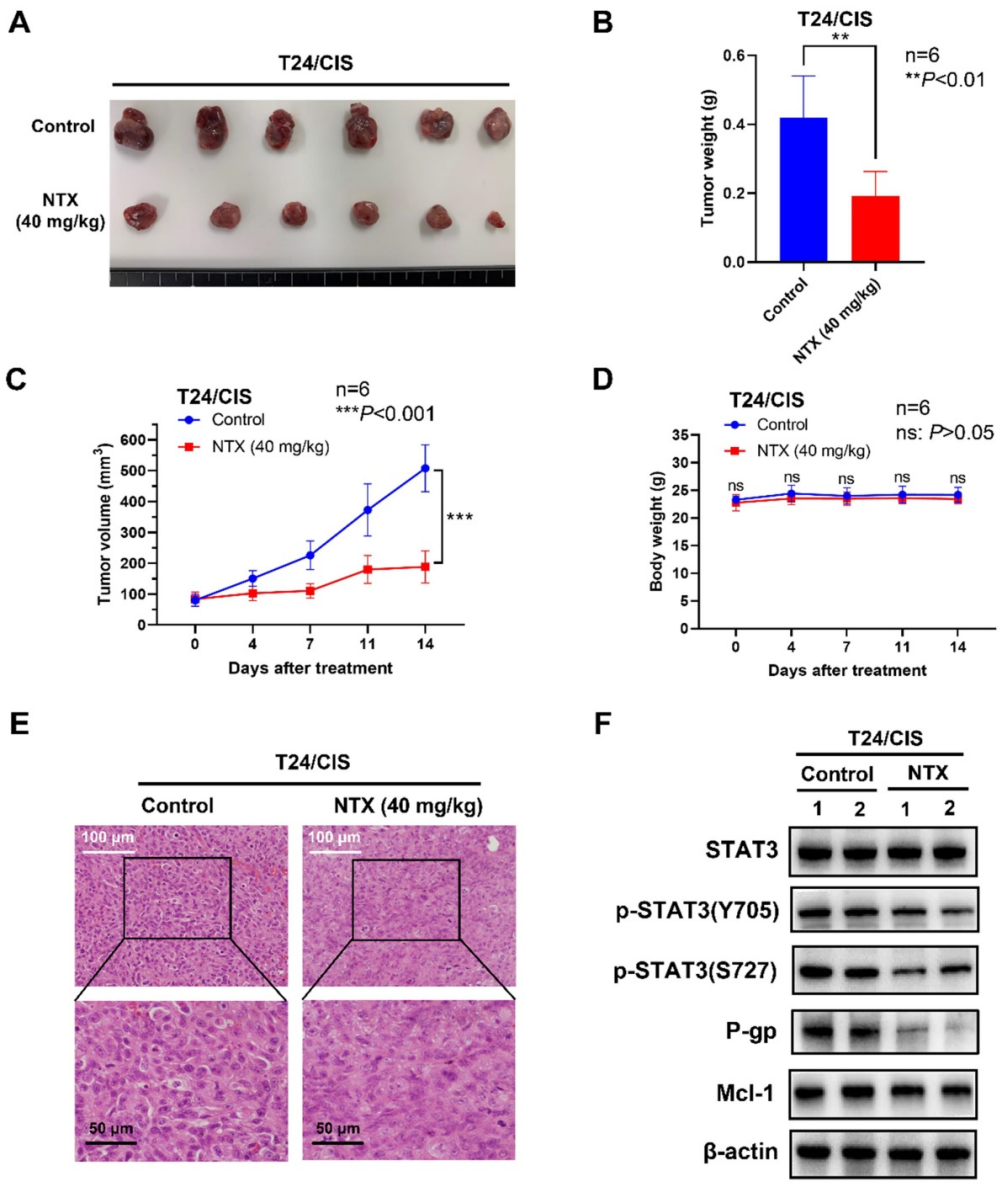
** NTX exhibits the anti-tumor effect on T24/CIS in vivo. (A)** T24/CIS tumors were excised from mice and photographed after two-week treatment.** (B)** The average tumor weight of NTX treatment group significantly decreased compared with that of vehicle group. **(C)** The growth curves of T24/CIS tumors at the indicated days following NTX treatment. **(D)** No significant difference was found in the body weight of T24/CIS tumor-bearing mice between treatment group and vehicle group. Data are expressed as the mean ± SD. **P < 0.01, ***P < 0.001, ns: *P* > 0.05, vs the vehicle group. **(E)** H&E staining was performed to observe the morphological alterations in T24/CIS tumors after NTX treatment. White scale bar = 100 μm; Black scale bar = 50 μm. **(F)** Proteins were extracted from T24/CIS tumors and western blot was used to detect the expressions of STAT3, p-STAT3 (Y705), p-STAT3 (S727), P-gp, and Mcl-1.

To determine NTX-induced anti-tumor effect in vivo, we established the T24/DOX and T24/CIS tumor xenograft models, followed by oral administration of NTX (40 mg/kg) or PBS (vehicle group). The representative changes of tumors were shown in **Figure 5A, 6A**, showing a significantly lower tumor size in NTX group than that in control group. The average tumor weights were (0.272±0.031) g for vehicle group and (0.095±0.022) g for NTX group in T24/DOX model (**Figure 5B**), while those were (0.420±0.121) g for vehicle group and (0.192±0.072) g for NTX group in T24/CIS model (**Figure 6B**). Similar for T24/DOX and T24/CIS models, the tumors grew slowly in NTX group when compared with the vehicle group (**Figure 5C, 6C**). No significant differences were found in the mean body weights of T24/DOX or T24/CIS tumor-bearing mice between NTX group and the corresponding control group (**Figure 5D, 6D**), indicating a low toxicity of NTX in vivo.

The morphological changes of H&E staining were shown in **Figure 5E, 6E**. The tumor cells in vehicle group were intact with deeply stained, large and abnormal nuclei, while those in NTX group presented with disappeared nuclei and tumor necrosis. Western blot analysis of T24/DOX and T24/CIS tumor tissues revealed that the expressions of p-STAT3 (Y705), p-STAT3 (S727), P-gp, and Mcl-1 were reduced by NTX treatment as compared to vehicle group, but there was no significant difference in STAT3 (**Figure 5F, 6F**).

## Discussion

The high rates of recurrence and progression require repeated cycles of chemotherapy for a long-term duration, and subsequently trigger frequent MDR in UBC, leading to poor prognosis and heavy economic burden 3, 5. In this study, NTX was firstly discovered as a STAT3 signaling inhibitor to trigger G0/G1 cell cycle arrest, apoptosis, and reverse P-gp overexpression in drug-resistant UBC. T24/DOX and T24/CIS cells have been well-established for decades to apply for basic research on UBC drug resistance to DOX or CIS 26-28. Following the confirmation of drug-resistant characteristics, we identified the overexpressions of STAT3 and P-gp in T24/DOX and T24/CIS cells. Moreover, we demonstrated by XTT assay that T24/DOX and T24/CIS cells were resistant to DOX and CIS, respectively, while both of them were sensitive to NTX time- and dose-dependently.

Recent studies have reported NTX arrests glioma and myeloma cells at G0/G1 stage 23, 29. Flow cytometric analysis also indicated NTX treatment led to the G0/G1 accumulation of DOX- and CIS-resistant T24 cells. Promoting cell cycle progression is a major carcinogenic mechanism of the proto-oncogene c-Myc, which potentially binds to Cyclin D1 promoter and upregulates its expression 30. As an allosteric regulator, Cyclin D1 forms a complex with cyclin-dependent kinase 4/6 (CDK4/6), and active complex (Cyclin D1-CDK4/CDK6) drives cell cycle transition from G1 to S phase 31. To explain the mechanism on NTX-induced G0/G1 arrest in T24/DOX and T24/CIS cells, we next demonstrated that NTX treatment downregulated c-Myc, Cyclin D1, CDK4, and CDK6.

Typical cellular changes, such as chromatin condensation, nuclear fragmentation, and even cell shrinkage, often accompany with the process of programmed cell death, apoptosis 32. Consistently, we observed these typical morphological changes in T24, T24/DOX, and T24/CIS cells following NTX treatment, which were further confirmed by flow cytometric analysis. Mitochondria-mediated apoptosis is crucially regulated by the B-cell lymphoma-2 (Bcl-2) family members, among which the anti-apoptotic ones such as myeloid cell leukemia 1 (Mcl-1), B-cell lymphoma-extra large (Bcl-xL) are well-validated anticancer targets 33. As a member of the inhibitor of apoptosis (IAP) family, Survivin is overexpressed in multiple cancers, and its upregulation in cancer is associated with chemoresistance and radioresistance 34. The anti-apoptotic protein expressions of Bcl-xL, Mcl-1, and Survivin could be negatively regulated by NTX treatment in T24/DOX and T24/CIS cells.

**Figure 7:**
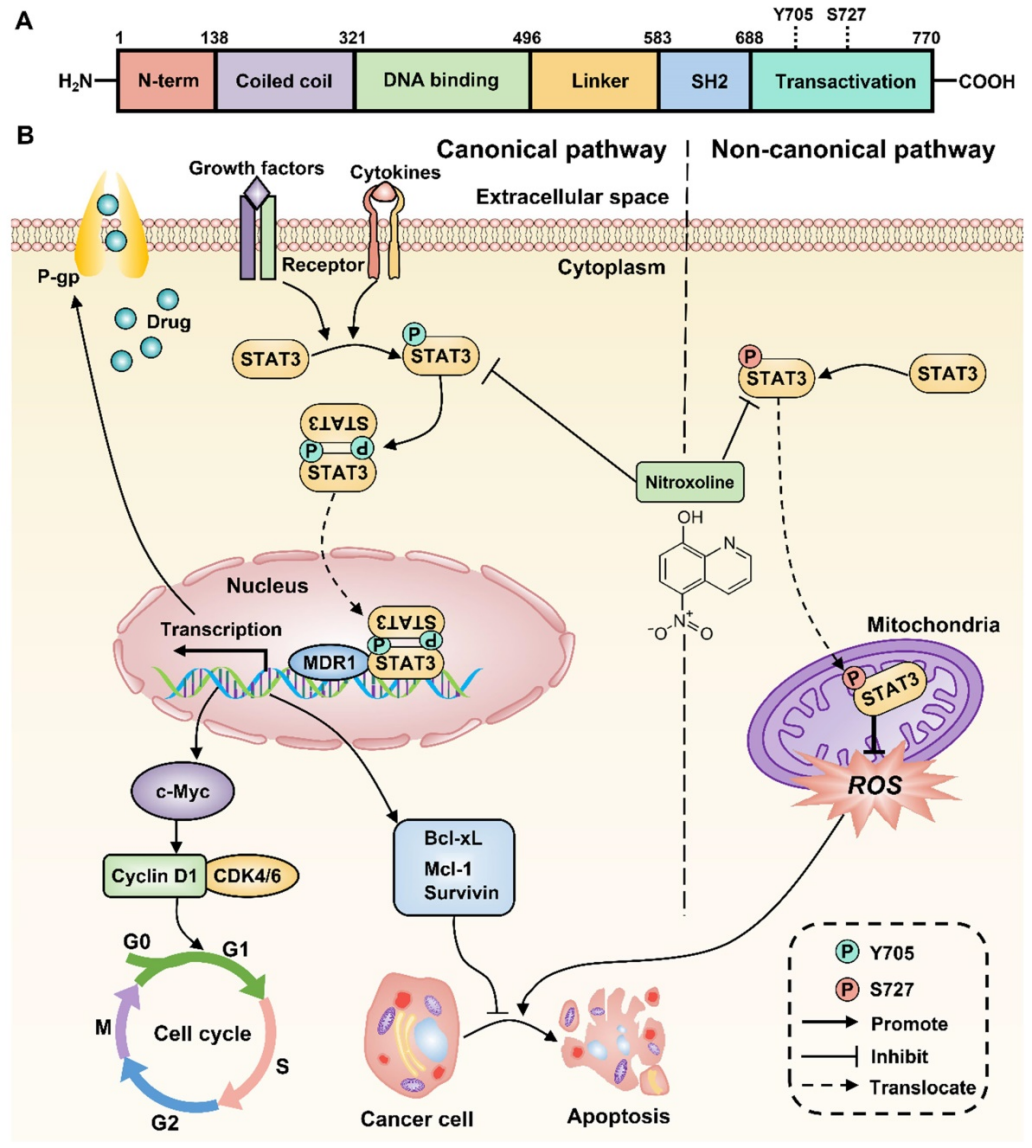
** Proposed mechanism for NTX-induced P-gp reversal, G0/G1 arrest and apoptosis in drug-resistant T24 cells.** The molecular structure of STAT3 is classic in STAT family, consisting of N-term Domain for cooperative DNA binding, Coiled-coil Domain for STAT3 recruitment to a receptor, DNA binding Domain, Linker Domain, SH2 Domain for STAT3 dimerization, and Transactivation Domain for transcription activation. **(B)** STAT3 can be activated to signal through both canonical and non-canonical pathways. For the canonical pathway, NTX inhibits STAT3 phosphorylation at Y705 residue and then decreases the translocation of STAT3 dimers to the nucleus in T24/DOX and T24/CIS cells. The subsequent downregulation of target genes including MDR1 gene, the cell cycle regulatory genes such as c-Myc, Cyclin D1, and the anti-apoptotic genes such as Survivin, Mcl-1, Bcl-xL, will reverse P-gp, trigger G0/G1 arrest, and induce apoptosis, respectively. For the non-canonical pathway, NTX-downregulated STAT3 phosphorylation at S727 residue reduces the translocation of p-STAT3 (S727) to the mitochondria and finally induces cell apoptosis by the increased generation of reactive oxygen species (ROS).

Increasing evidence suggests the critical role of STAT3 in chemoresistance, proliferation, and apoptosis of multiple tumor cells 11. Previous studies have also reported that STAT3 overexpression occurs in drug-resistant UBC cell lines and associates with poor prognosis of UBC patients 12, 13. Therefore, inhibition of STAT3 signaling is a promising therapeutic strategy to decrease chemoresistance and induce apoptosis of drug-resistant UBC. Consistently, the expressions of STAT3 and P-gp were upregulated in T24/DOX and T24/CIS cells compared with T24 parental cells. After membrane surface receptors are stimulated by cytokines or growth factors, STAT3 can be activated to signal through both canonical and non-canonical pathways 35. For the canonical pathway, the subsequent phosphorylation of STAT3 at Y705 residue will homodimerize or heterodimerize through their SH2 domains (**Figure 7A**). Following nuclear translocation, STAT3 dimers can bind to promoter elements of target genes including the MDR1 gene, the cell cycle regulatory genes such as c-Myc, Cyclin D1, and the anti-apoptotic genes such as Survivin, Mcl-1, Bcl-xL, and further modulate their transcription 36. For the non-canonical pathway, the phosphorylation of STAT3 at S727 residue is required for maximal STAT3 activation 14. The subsequent translocation of p-STAT3 (S727) to the mitochondria promotes tumor cell proliferation by reducing the production of reactive oxygen species (ROS), a mediator of cell apoptosis 37. In this study, NTX was identified as a potent STAT3 inhibitor to downregulate the expressions of STAT3, p-STAT3 (Y705), and p-STAT3 (S727) in T24/DOX and T24/CIS cells. The molecular-level explanation for NTX causing P-gp reversal, G0/G1 arrest, and apoptosis in T24/DOX and T24/CIS cells, is presented in **Figure 7B**. NTX treatment downregulated the expressions of STAT3 and p-STAT3 (Y705), which subsequently decreased the translocation of STAT3 dimers into the nucleus. Then, the downstream targets, including MDR1, c-Myc, Cyclin D1, Survivin, Mcl-1, and Bcl-xL, were downregulated, which could reverse MDR, trigger G0/G1 arrest, and induce apoptosis, respectively. Moreover, the downregulated p-STAT3 (S727) could promote ROS generation, which further mediated the apoptosis of T24/DOX and T24/CIS cells. According to the recent reports, there is still a huge controversy on whether p-STAT3 (S727) regulates p-STAT3 (Y705) negatively or positively 38-40, which needs further experimental confirmation. NTX and Stattic, a selective inhibitor of STAT3 activation, dimerization, and nuclear translocation 41, synergistically decreased p-STAT3 (Y705), P-gp, and the anti-apoptotic member Mcl-1, which further verified NTX-mediated STAT3 inhibition in drug-resistant UBC.

The two-week NTX treatment (40 mg/kg/day) also significantly in vivo inhibited T24/DOX and T24/CIS tumor growth. The dosage was selected based on the recommended dose for urinary tract infections 42, the guide for dose conversion between animals and human 43, and animal experiments in previous studies 20, 21. The mean body weights of T24/DOX or T24/CIS tumor-bearing mice exhibited no between-group difference, which also indicated its favorable safety profile. Interestingly, by H&E staining, we also observed the disappeared nuclei and tumor necrosis in NTX group.

To our knowledge, this is the first report to discover NTX as a STAT3 inhibitor for drug-resistant UBC therapy. However, it should be noted that single agent therapy for UBC potentially leads to chemotherapy failure with limited therapeutic efficacy and drug resistance 44. It remains to be investigated whether NTX or its analogues combined with DOX or CIS can enhance the anti-tumor effect, promote the extrusion of drugs into extracellular space by drug efflux pumps, and even delay the development of drug resistance in UBC without significantly increasing toxicity. We also noticed that previous clinical trials focused on the therapeutic effect of NTX (APL-1202) on NMIBC, while a multicenter study (NCT04813107, Phase I/II) on the combination of NTX with tislelizumab, a monoclonal antibody against programmed cell death protein 1 (PD-1) 45 will be started to assess their efficacy and safety in cisplatin-ineligible MIBC patients. Our previous work found that NTX decreases the levels of myeloid-derived suppressor cells (MDSCs) in UBC-bearing murine model 20. Interestingly, Takeyama et al. recently reported the elevated MDSCs infiltration in CIS-resistant UBC microenvironment 46. Further studies are expected to verify the combined efficacy of NTX and PD-1 antibody, and whether NTX could potently inhibit drug-resistant UBC through targeting MDSCs.

## Conclusions

Here we reported that NTX as a STAT3 inhibitor exhibits the significant efficacy against drug-resistant UBC. Our observations demonstrated that NTX induces P-gp reversal, G0/G1 arrest, and apoptosis in drug-resistant UBC via suppression of the STAT3 signaling pathway. Our findings could provide a molecular-level basis in repurposing NTX with clinical implications against drug-resistant UBC by targeting STAT3 signaling.

## Figures and Tables

**Table 1:** IC50 values* of DOX, CIS, or NTX determined in T24, T24/DOX and T24/CIS cells.

	T24		T24/DOX		T24/Cis	
	IC50 (μM)	95%CI	IC50 (μM)	95%CI	IC50 (μM)	95%CI
DOX (24 h)	0.71	0.56-0.89	10.93	6.88-17.55	-	-
CIS (24 h)	16.27	13.29-19.11	-	-	58.04	51.11-67.55
NTX (24 h)	15.22	12.77-18.19	14.91	12.99-17.02	19.72	18.11-21.53
NTX (48 h)	7.85	7.15-8.61	10.69	9.31-12.27	11.20	10.17-12.30
NTX (72 h)	4.48	4.17-4.82	7.32	6.55-8.16	9.15	8.09-10.27

*IC50 values represent the concentrations of DOX, CIS, or NTX producing 50% cell growth inhibition. Abbreviations: IC50, half maximal inhibitory concentration; 95%CI: 95% confidence interval; DOX: doxorubicin; CIS: cisplatin; NTX: nitroxoline.

## Abbreviations

UBC: urothelial bladder cancer
NTX: nitroxoline
MIBC: muscle-invasive bladder cancer
DOX/ADM/ADR: doxorubicin
CIS/DDP/CDDP: cisplatin
P-gp: P-glycoprotein
MDR: multidrug resistance
ABCB1: ATP-binding cassette B1
MDR1 gene: multidrug resistance 1 gene
ChIP: chromatin immunoprecipitation
STAT3: Signal transducer and activators of transcription 3
MetAP2: methionine aminopeptidase-2
PD-1: programmed cell death protein 1
H&E staining: hematoxylin and eosin staining
IC50: half maximal inhibitory concentration
95%CI: 95% confidence interval
CDK4/6: cyclin-dependent kinase 4/6
Mcl-1: myeloid cell leukemia 1
ROS: reactive oxygen species
MDSCs: myeloid-derived suppressor cells
ANOVA: one-way analysis of variance
Bcl-xL: B-cell lymphoma-extra large
